# Adipokines: Potential Therapeutic Targets for Vascular Dysfunction in Type II Diabetes Mellitus and Obesity

**DOI:** 10.1155/2017/8095926

**Published:** 2017-02-13

**Authors:** Mostafa Wanees Ahmed El husseny, Mediana Mamdouh, Sara Shaban, Abdelrahman Ibrahim Abushouk, Marwa Mostafa Mohamed Zaki, Osama M. Ahmed, Mohamed M. Abdel-Daim

**Affiliations:** ^1^Faculty of Medicine, Fayoum University, Fayoum, Egypt; ^2^NovaMed Medical Research Association, Cairo, Egypt; ^3^Fayoum Medical Student Association, Fayoum, Egypt; ^4^Faculty of Medicine, Ain Shams University, Cairo, Egypt; ^5^Faculty of Clinical Pharmacy, Fayoum University, Fayoum, Egypt; ^6^Physiology Division, Zoology Department, Faculty of Science, Beni-Suef University, Beni-Suef, Egypt; ^7^Pharmacology Department, Faculty of Veterinary Medicine, Suez Canal University, Ismailia, 41522, Egypt

## Abstract

Adipokines are bioactive molecules that regulate several physiological functions such as energy balance, insulin sensitization, appetite regulation, inflammatory response, and vascular homeostasis. They include proinflammatory cytokines such as adipocyte fatty acid binding protein (A-FABP) and anti-inflammatory cytokines such as adiponectin, as well as vasodilator and vasoconstrictor molecules. In obesity and type II diabetes mellitus (DM), insulin resistance causes impairment of the endocrine function of the perivascular adipose tissue, an imbalance in the secretion of vasoconstrictor and vasodilator molecules, and an increased production of reactive oxygen species. Recent studies have shown that targeting plasma levels of adipokines or the expression of their receptors can increase insulin sensitivity, improve vascular function, and reduce the risk of cardiovascular morbidity and mortality. Several reviews have discussed the potential of adipokines as therapeutic targets for type II DM and obesity; however, this review is the first to focus on their therapeutic potential for vascular dysfunction in type II DM and obesity.

## 1. Introduction

According to the World Health Organization (WHO) reports, obesity affects 1.1 billion adult individuals worldwide, 300 millions of whom are clinically obese with a body mass index (BMI) of ≥ 30 kg/m^2^ [1, 2]. It can cause several health problems, including hypertension, stroke, and ischemic heart disease [1]. An analysis by the Prospective Studies Collaboration (PSC) of data from over 900,000 individuals showed that the median survival rate decreases by 8 to 10 years for obese subjects with a BMI of 40 to 45 kg/m^2^, compared to those with a normal BMI, primarily due to vascular complications [3].

Obesity and type II diabetes mellitus (DM) are linked because both are components of the so-called “metabolic syndrome (MS).” Metabolic syndrome is defined by the WHO as the presence of insulin resistance (IR) and any two of the following criteria which include (1) increased abdominal girth, (2) hypertension, (3) elevated triglycerides levels, (4) low high-density lipoprotein level, and (5) elevated blood glucose levels (type II DM) [4]. It is now considered a major health problem with a prevalence of 34% among the United States population, and it has a significant association with increased risks of morbidity and mortality from cardiovascular diseases [5].

Adipose tissue is no longer considered a passive site for storage of energy in the form of triacylglycerols [6], but it is also an important endocrine gland which secretes several bioactive molecules [7–10]. Adipokines (or adipocytokines) are bioactive molecules that regulate several physiological functions such as energy balance, insulin sensitization, appetite regulation, inflammatory response, and vascular homeostasis [11, 12]. They are primarily secreted by adipose tissue cells (adipocytes), but recent studies found that intestinal epithelium, adrenal gland, skeletal muscle, leukocytes, macrophages, hepatocytes, and cardiomyocytes can secrete them as well [2, 13].

All blood vessels are surrounded by a layer of perivascular adipose tissue (PVAT), in which adipocytes are encroaching into the adventitia of the vessel, permitting secreted factors from PVAT to enter the blood circulation instantly [14–16]. This tissue is involved in vascular inflammation, adjustment of vascular tone, and smooth muscle cells' proliferation [17]. It can promote endothelium-dependent vasodilatation by releasing nitric oxide (NO), adiponectin, and adipocyte derived relaxing factor (ADRF), which activate potassium channels of the vascular smooth muscle cells and thus inhibit the vasoconstrictive effect of multiple agonists including phenylephrine, serotonin, and angiotensin II [14, 16]. On the other hand, PVAT secretes adipocyte fatty acid binding protein (A-FABP) and adipose tissue-derived constricting factor (ADCF), which induce vasoconstriction and activate NADPH oxidase enzyme, increasing the release of reactive oxygen species (ROS) [17].

In obese and type II diabetics, insulin resistance causes impairment of the endocrine function of PVAT, an imbalance in the secretion of vasoconstrictor and vasodilator molecules, and increasing of the production of ROS, all of which lead to endothelial dysfunction and vascular hypertension [2]. A recent study has shown that adiponectin needs to be 10 times higher to mediate the same vasodilator effect in obese individuals, compared to normal controls [18].

Moreover, increased systemic and vascular oxidative stress in these patients directly inhibits the release of NO, which mediates vasodilatation and protects the arterial wall from atherogenic mediators, leading to endothelial dysfunction and a proatherogenic environment [19]. Several studies have shown that adipose tissue inflammation promotes the hepatic production of C-reactive protein (CRP) and fibrinogen, which stimulate the expression of adhesion molecules such as vascular cell adhesion molecule-1 (VCAM-1) and intercellular adhesion molecule-1 (ICAM-1), creating a prothrombotic state, which gradually progresses to cardiovascular disease [11, 12, 20, 21].

Several reviews have discussed the potential of adipokines as therapeutic targets for type II DM and obesity [22, 23]; however, this review is the first to focus on their therapeutic potential for vascular dysfunction in type II DM and obesity. This article explores the vascular functions of different adipokines, how these functions are impaired in insulin resistance states, and how targeting adipokines can serve as a therapeutic strategy for these abnormalities.

## 2. Vascular Effects of Adipokines

### 2.1. Adiponectin

Adiponectin is an insulin-sensitizing adipokine, involved in glucose and lipid homeostasis, through activation of AMP-activated protein kinase (AMPK) [24, 25]. It works through its receptors, including AdipoR1 (skeletal muscle), AdipoR2 (liver), and T-cadherin (cardiovascular system) [2]. In the circulation, adiponectin is present as three distinct oligomeric complexes, and it was found that the distribution, not the absolute amount of total adiponectin, determines insulin sensitivity [26, 27]. In skeletal muscles, it decreases lipid accumulation by promoting fatty acid *β*-oxidation and increasing glucose uptake [28, 29]. In the liver, it inhibits both gluconeogenesis and lipogenesis, counteracting hyperglycemia and hepatic steatosis [30, 31]. Unlike most adipokines, adiponectin is inversely proportionate to the degree of obesity, meaning that its concentration decreases when obesity becomes more severe and is restored when the body weight is reduced [32].

In terms of vascular functions, adiponectin has several anti-inflammatory and endothelium-protective effects. It induces downregulation of the myelomonocytic precursor cells, inhibits the phagocytic activity of mature macrophages, and reduces the release of proinflammatory cytokines, including tumor necrosis factor-alpha (TNF-*α*), interferon-gamma (IFN-*γ*), interleukin-10 (IL-10), and IL-1 receptor antigen (IL-1RA) [33]. It inhibits leukocyte-endothelium reaction, which is a major risk factor for both macrovascular and microvascular complications [34].

In case of vascular endothelial damage or tissue ischemia, it promotes migration of endothelial progenitor cells (EPCs) towards the area of vascular damage, where they turn into mature endothelial cells. Therefore, in humans, plasma levels of adiponectin are directly proportionate with the number of circulating EPCs [35]. Furthermore, animal studies have shown impaired repair of vascular injury in adiponectin deficient mice with deficient recruitment of ECPs from the bone marrow to the site of injury [36].

On the cellular endothelial level, it reduces production of ROS, usually caused by elevated glucose and low-density lipoprotein (LDL) levels, through inhibition of the NAD(P)H oxidase enzyme [37]. Clinical studies in humans showed that increased plasma levels of adiponectin are associated with diminished markers of oxidative stress [38]. Animal studies have found that adiponectin suppresses the activation of nuclear factor-kappa B (NF-kB) in endothelial cells, through a protein kinase A (PKA) dependent pathway [39]. Other studies have indicated that adiponectin suppresses apoptosis and caspase-3 activity in human umbilical vein endothelial cells (HUVECs) through activation of the AMPK signaling pathway, and it prevents angiotensin II-induced apoptosis of bovine endothelial cells by restoring eNOS-HSP90 reaction [24, 40].

Moreover, adiponectin has an antiatherogenic role through inhibition of smooth muscle cells' proliferation and migration [41], which are induced by atherogenic growth factors, including heparin-binding epidermal growth factor, platelet-derived growth factor- (PDGF-) BB, and basic fibroblast growth factor [38, 42]. Kubota et al. reported excessive smooth muscle migration and proliferation with neointimal hyperplasia in mice deficient of adiponectin [43]. Moreover, it was shown that patients with angina pectoris with lower levels of adiponectin are at a high risk for progression of their atherosclerotic lesions with development of acute coronary syndrome [44]. Adiponectin guards against myocardial ischemia/reperfusion (I/R) injury by inhibiting the production of local TNF-*α* and cardiomyocyte apoptosis that are induced by I/R injury [45]. It also alleviates monocyte attachment to endothelial cells through suppression of resistin-induced expression of adhesion molecules [40].

Different mechanisms link obesity-associated hypertension to decreased levels of adiponectin including hyperactivity of the renin-angiotensin system and sympathetic nervous system, endothelial dysfunction, and natriuresis impairment [46]. A recent study estimated that each 1 mg/mL increase in adiponectin levels was associated with a 6% reduced risk of hypertension [47].

### 2.2. Adipocyte Fatty Acid Binding Protein (A-FABP)

Adipocyte fatty acid binding protein, also termed FABP-4, is a transport protein that delivers its endogenous ligands, including oleic acid, retinoic acid, and arachidonic acid to the nuclear receptor [peroxisome proliferator-activated receptor-*γ* (PPAR-*γ*)], thus encouraging the transcriptional activity of the receptor, which plays a major role in lipolysis [48]. It is mainly expressed in adipocytes, but it can be also produced from macrophages. Its level in human plasma ranges from 10 to 50 ng/mL, a level that is much higher than other adipokines [49]. Elevated plasma levels of A-FABP can serve as a marker for several obesity-related metabolic abnormalities, endothelial dysfunction, hypertension, atherosclerosis, and coronary heart disease [48]. It was found that A-FABP increases as coronary heart disease progresses from one vessel to three-vessel disease [50]. Its serum level is directly proportionate with adiposity measures (BMI, fat percentage, and waist circumference) and serum levels of lipocalin-2 and hsCRP, which are two inflammatory markers associated with atherosclerosis and coronary artery disease [51]. However, it is inversely correlated with the serum levels of adiponectin. Built upon these findings, A-FABP is a proinflammatory cytokine that links obesity to vascular dysfunction.

The expression of A-FABP in macrophages can be triggered by saturated free fatty acids, oxidized LDL, and Toll-like receptor activators. Such expression causes intracellular accumulation of cholesterol-ester and formation of foam cells, while inhibiting A-FABP expression increases cholesterol efflux and inhibits the activity of NF-kB and cyclooxygenase-II enzyme. Another proinflammatory mechanism of A-FABP is its positive feedback on C-Jun N-terminal kinase (JNK), a major mediator of insulin resistance and vascular dysfunction. This means that inflammatory stimuli activate JNK in macrophages, which enhance gene transcription of A-FABP. Conversely, elevated A-FABP levels increase JNK phosphorylation, leading to its activation and increased levels of proinflammatory cytokines [52].

### 2.3. Leptin

Leptin is another adipokine which regulates food intake by inducing satiety and facilitating energy expenditure. Its serum levels are directly proportionate with the body adipose mass and adipocyte size. Clinical studies showed a positive correlation between leptin levels and development of macrovascular complication of obesity such as myocardial infarction and cerebral stroke [53, 54]. A large prospective study on leptin and cardiovascular risk (West of Scotland Coronary Prevention Study) highlighted leptin as an independent predictor of coronary events such as cardiac death, myocardial infarction, and coronary revascularization with a great prognostic value in atherosclerotic patients [55]. Another application for these clinical observations is the use of leptin-adiponectin ratio as an indicator of subclinical atherosclerosis because it correlates with the magnitude of the intima-media thickness (IMT) of common carotid artery. A recent study showed increasing leptin expression with diminished adiponectin levels in patients with coronary artery disease [56].

The association of leptin to hypertension is an interesting point of controversy [57]. Under physiological conditions, leptin induces endothelial NO production with subsequent vasodilation [58]. However, in cases of obesity-associated hyperleptinemia, this vasodilatory effect is lost with subsequent development of hypertension. The controversy upon the peripheral effect of leptin supports the proposed theory of an involved central mechanism. Leptin, through its central receptors, has a stimulatory effect on the sympathetic nervous system with subsequent elevation of blood pressure in cases of hyperleptinemia [59]. Human leptin deficiency syndrome is associated with severe obesity and hypotension that can be explained by lack of its central effect on satiety centre and sympathetic nervous system [60].

The role of leptin as a prothrombotic factor in the pathogenesis of cardiovascular disease is now well established. Animal studies have shown that leptin can increase platelet aggregation with subsequent arterial thrombosis [61, 62]. Furthermore, leptin at high concentrations can induce expression of vascular adhesion molecules and the prothrombotic tissue factor [63]. Clinical studies have shown a positive correlation between plasma levels of leptin and plasminogen activator inhibitor-1 (PAI-1), tissue plasminogen activator, von Willebrand factor (vWF), and fibrinogen [63]. Leptin also promotes the expression of other inflammatory adipocytokines including TNF-*α*, IL-2, IL-6, and monocyte chemotactic protein 1 (MCP-1) that have a prothrombotic effect. Another study has shown that leptin stimulates the expression of C-reactive protein (CRP) in coronary endothelial cells [64], creating a prothrombotic state in the coronary circulation [65].

### 2.4. Chemerin

Chemerin is a novel adipokine that is highly expressed in adipose tissue, liver, and cells of innate immunity [66, 67]. Recently, there has been a great interest in its potential role in the pathogenesis of cardiovascular disease in patients with MS. Several observational studies reported elevated levels of chemerin in obese patients [68, 69]. A recent study (2015) has investigated the role of chemerin in endothelial cell activation and early atherosclerotic changes in patients with newly diagnosed type II DM and showed that chemerin can increase the expression of endothelial adhesion molecules (ICAM-1 and E-selectin) that share in the early steps of forming an atherosclerotic plaque [6].

### 2.5. Omentin

Omentin is a recently discovered adipokine that is produced primarily by PVAT and vascular stromal cells [63]. Metabolically, omentin increases insulin-induced cellular signaling with promotion of insulin sensitivity within the adipose tissue. An inverse correlation exists between omentin levels and adiposity measures, including BMI and waist circumference [70]. Human studies reported diminished omentin levels in patients with severe coronary atherosclerosis, highlighting its role in preventing arterial calcification, which contributes to the development of atherosclerosis [71–75]. This antiatherogenic role is potentiated by its inhibitory effect on smooth muscle migration and proliferation [76]. Omentin has a NO-mediated vasodilator effect that can antagonize TNF-*α* mediated vasoconstriction [63]. Moreover, it inhibits expression of adhesion molecules such as E-selectin on endothelial cells, which play an important role in early stages of atherosclerosis [77].

### 2.6. Resistin

Human resistin is a 108 amino acid, cysteine-rich protein, encoded by a gene located on chromosome 19 [78]. It is a proinflammatory adipokine that is involved in the mechanism of IR, an action that is highly linked to the levels of other inflammatory adipocytokines such as leptin, TNF-*α*, and IL-6 [79–82]. Recent studies showed the presence of resistin-leptin cross-talk that regulates glucose metabolism and energy regulation [83].

Recent clinical studies indicated a positive correlation between high plasma levels of resistin and the severity of unstable angina (UA), atherosclerosis, and poor prognostic cases of coronary artery disease [84, 85]. Furthermore, patients with acute coronary syndrome (ACS) showed high levels of resistin with enhanced resistin gene expression within the adipose tissue, in comparison to cases of stable angina [86]. Experimental evidence supports the role of resistin in the pathogenesis of cardiovascular disease. Verma highlighted the potential role of resistin in endothelial dysfunction [87]. Other studies reported that resistin increased the release of endothelin, an effective vasoconstrictor in endothelial cells, and prothrombotic atherogenic factors such as plasminogen activator inhibitor-1 (PAI-1) and vWF that inhibit NO production [87, 88]. Moreover, resistin promotes smooth muscle cells proliferation and migration [89, 90] and increases the synthesis of prothrombotic tissue factor (TF) in human coronary cells [91].

### 2.7. Other Adipokines

Adipsin is a potential indicator of obesity in rodents. Although its role in energy homeostasis and systemic metabolism remains unknown, higher serum adipsin levels are found in diabetic and hypertensive patients [92]. Visfatin or PBEF (pre-B cell colony-enhancing factor) is involved in glucose uptake and metabolism because it stimulates insulin receptors [93]. It is also a prognostic factor of cardiovascular mortality because it plays a significant role in plaque destabilization of unstable carotid and coronary atherosclerosis [94].

Apelin (APE) is another adipokine that was known as the endogenous ligand of the orphan G-protein-coupled receptor. Elevated levels of apelin in obese mice reflect mild obesity-related inflammation, characterized by an increase in macrocytic count and high TNF-*α* levels. APE has a positive inotropic effect on the heart. Now, it is believed to be an angiotensin II homologue, performing beneficial effects on the aortic wall, causing its relaxation when used as a treatment [94].

**Figure 1:**
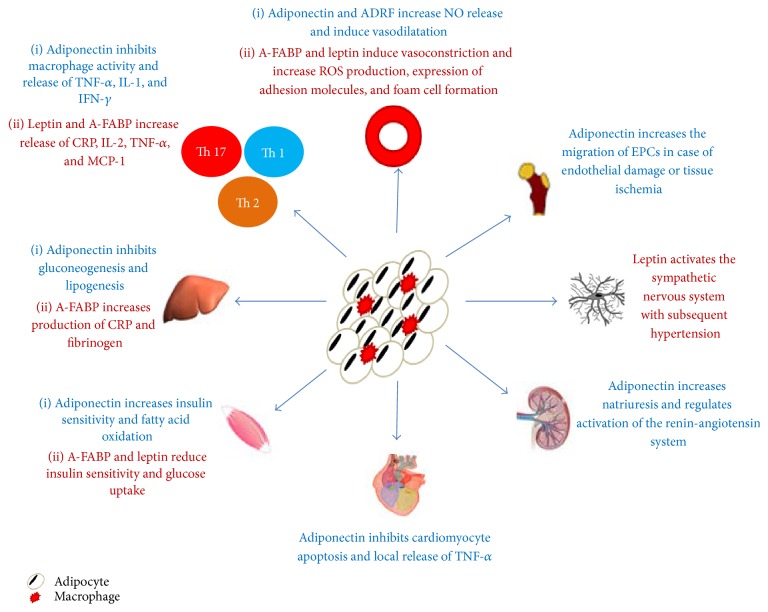
The figure summarizes the direct and indirect effects of different adipokines on the vascular system.

Another proinflammatory adipocytokine is retinol binding protein-4 (RBP-4). Overexpression of RBP-4 triggers adipose tissue inflammation through the stimulation of both the innate and adaptive arms of the immune response [95]. RBP4 directly stimulates antigen-presenting cells, activating CD4+ T-helper cells, which trigger adipose tissue inflammation and, therefore, insulin resistance [96]. These data indicate that decreasing RBP4 levels, utilizing fenretinide or thiazolidinedione, constitutes a promising therapeutic strategy to improve insulin resistance; however, this mechanism has not been investigated in human trials [22]. The vascular effects of different adipokines are summarized in Figure 1.

## 3. Adipokines as Therapeutic Targets for Vascular Dysfunction

Based on the aforementioned vascular roles, several therapeutic strategies have been proposed and tested to improve vascular dysfunction in obese individuals and type II diabetics, including the following.

### 3.1. Increasing Adiponectin Activity

Several drugs with antidiabetic activity (glimepiride, peroxisome proliferator-activated receptor-alpha [PPAR*α*] agonists: thiazolidinediones) [97], renin-angiotensin system (RAS) blocking effect such as losartan [98–100], and cholesterol/triglycerides lowering effect such as simvastatin or fenofibrate have been shown to increase adiponectin plasma levels in human and animal studies [101]. Fenofibrate therapy for two months markedly elevated plasma adiponectin levels and insulin sensitivity in patients with primary hypertriglyceridemia [101, 102]. Moreover, high plasma levels of adiponectin were noticed in humans receiving dietary fish oil [103]. Natural substances such as astragaloside II and isoastragaloside I (extracted from the medicinal herb Radix-Astragali) were shown to increase adiponectin plasma levels and reduce obesity-related insulin resistance [104].

Direct pharmacological administration of adiponectin has been shown to reduce glucose, lipid, and insulin concentrations and increase insulin receptor expression in obese diabetic mice on high fat diet [105]. A study by Kase et al. (2007) reported that treatment with globular adiponectin stimulated NF-*κ*B of activated B cells and increased the expression of cohesive molecules and MCP-1 in endothelial cells by stimulation of the sphingosine kinase signaling pathway [106].

Lately, an orally active adiponectin receptor agonist “adipoRon” was proven to ameliorate insulin resistance and glucose intolerance in mice [22, 107]. An in vitro study has shown that osmotin, one of the pathogenesis related-5 (PR-5) family of plant defense proteins, is a potential adiponectin receptor agonist [108]. The three-dimensional structure of osmotin is similar to globular adiponectin; both are antiparallel *β*-strands arranged in the shape of a *β*-barrel. In addition, osmotin stimulates AMPK pathway via adiponectin receptors in mammalian C2C12 myocytes [97].

Repressed expression of the two adiponectin receptors (AdipoR1/AdipoR2) and hypoadiponectinemia have been shown in patients with type II DM [109] and in obese animals with insulin resistance and vascular dysfunction [110]. Therefore, enhancing AdipoR1/AdipoR2 expression or developing receptor agonists that can mimic adiponectin effects is a possible strategy to treat obesity-related vascular diseases [97]. Recent studies have shown that exercise, associated with a hypocaloric diet, increased AdipoR1 and AdipoR2 expression in skeletal muscle cells in older obese adults (>60 years of age) [23, 111]. In human macrophages, both PPAR*α* and PPAR*γ* agonists raised AdipoR2 expression only, while a synthetic LXR (liver X receptor) agonist activated the expression of both AdipoR1 and AdipoR2 [112].

### 3.2. Inhibiting A-FABP Activity

Based on the proinflammatory effects of A-FABP, pharmacological agents that inhibit its function may be a novel strategy for prevention of vascular dysfunction [113]. Animal studies have shown that pharmacologically induced reduction of A-FABP expression in apoE^−/−^ mice on high fat diet markedly reduced aortic atherosclerotic lesions. Boord et al. reported that A-FABP null mice had a 67% higher survival rate, compared to control mice when both groups received high fat diet for one year [114].

Several A-FABP repressors such as arbazole-based repressors, benzylamino-6-(trifluoromethyl)pyrimidin-4(1H) repressors, and BMS309403 have been identified [1, 115]. BMS309403 interacts with the fatty acid binding pocket of A-FABP to antagonize binding of endogenous fatty acids [116]. Animal trials showed that treatment with BMS309403 reduces C-Jun NH2-terminal kinase (JNK) stimulation and production of proinflammatory cytokines [113]. An animal study by Furuhashi et al. showed that oral administration of BMS309403 reduced atherosclerotic lesions and enhanced insulin sensitivity and glucose tolerance in apoE^−/−^ mice [117]. Until now, these effects of BMS309403 have not been tested in humans and further clinical trials are required before clinical development of this therapeutic agent.

### 3.3. The Relation between Adipokines and Anti-Inflammatory Drugs

The evidence of involvement of inflammatory pathways is confirmed by the protective activity of some anti-inflammatory drugs against obesity-related insulin resistance [118]. Aspirin can suppress not only JNK [119, 120], but other serine/threonine (Ser/Thr) protein kinases (mammalian target of rapamycin and protein kinase B/Akt) that induce IR by phosphorylation of insulin receptor substrate-1 (IRS-1) at serine residues [120]. Moreover, aspirin has been reported to inhibit the functionality of NF-*κ*B or activator protein-1 (AP-1) which are associated with increased production of ROS [121]. Salicylates treatment has been proven to ameliorate acute insulin resistance in genetically obese rodents [118, 122]. Moreover, pretreatment of rats with salicylates protected against lipid-activated insulin resistance in skeletal muscles by suppressing IRS-1 tyrosine phosphorylation and accompanied phosphatidylinositide 3-kinase (PI-3K) stimulation [123]. Treatment with high doses of aspirin (7 g/day) or salicylates (3 g/day) ameliorated peripheral insulin sensitivity in type II DM patients [124].  Table 1 summarizes the vascular functions and diagnostic or therapeutic implications of different adipokines.

## 4. Future Research and Clinical Development of Adipokines

The pharmaceutical development of adipokines or adipokine inhibitor drugs for clinical use may face multiple obstacles, including lack of funding, high development cost, legislation, and licensure regulations [125]. Moreover, lack of human data on the safety and efficacy of many adipokines and deficient understanding of their mechanism of action can block further development of adipokine-based therapeutic strategies [126]. In the past 15 years, only leptin was developed into a drug following its discovery [127, 128] and the role of dipeptidyl peptidase-4 (DPP-4) as an adipokine was understood after it has been used for treatment of type II DM [129, 130]. Future research should focus on identifying the basic mechanism of action for several adipokines such as chemerin, resistin, and apelin. The drug development process takes 15 years on average: four to five years in the preclinical phase and then about eight years in clinical trials, and then the legislation and drug approval process start [131]. Innovative approaches should be followed to shorten this period, especially in the legislation step [126]. The potential role of adipokine-based therapies for vascular dysfunction, type II DM, and obesity, as well as neuroinflammatory diseases should be advocated and shared with the public and practicing physicians [132, 133].

## 5. Conclusion

Adipokines are bioactive molecules that have different effects on the vascular system. In obese and type II diabetics, IR causes dysfunction of the endocrine function of PVAT, an imbalance in the secretion of vasoconstrictor and vasodilator molecules, and increased production of ROS. Recent studies have shown that targeting plasma levels of adipokines or the expression of their receptors can increase insulin sensitivity, improve vascular function, and reduce the risk of cardiovascular morbidity and mortality. However, several human trials are required before further clinical development of these strategies. Further resources should be allocated to basic research to enhance our understanding of the vascular roles of adipokines and develop new therapeutic strategies to target these roles.

## Figures and Tables

**Table 1:** The table shows the specific vascular functions of different adipokines and their roles as therapeutic targets for vascular dysfunction in type II DM and obesity.

Adipokine	Vascular effect	Diagnostic/therapeutic implications
Adiponectin	(i) Anti-inflammatory and endothelium-protective effects. (ii) Promoting repair of endothelial damage and tissue ischemia. (iii) Inhibition of smooth muscle cells' proliferation and migration. (iv) Reducing oxidative stress and associated cellular apoptosis.	(i) Increasing its plasma concentration by thiazolidinediones, RAS blockers, and cholesterol-lowering drugs. (ii) Direct pharmacological administration of adiponectin. (iii) Use of adiponectin agonists such as adipoRon or osmotin. (iv) Enhancing adiponectin receptor expression by hypocaloric diet, exercise, or pharmaceutical agents.

A-FABP	(i) Accumulation of cholesterol-ester within macrophages and foam cell formation. (ii) Positive feedback on C-Jun N-terminal kinase (JNK), a major mediator of insulin resistance and vascular dysfunction.	Using A-FABP pharmacological repressors such as arbazole-based repressors, benzylamino-6-(trifluoromethyl)pyrimidin-4(1H) repressors, and BMS309403.

Leptin	(i) At low levels, it induces hypotension and at high levels, it stimulates the sympathetic nervous system causing vasoconstriction and hypertension. (ii) At high levels, it increases platelet aggregation, expression of vascular adhesion molecules, and prothrombotic tissue factor.	(i) Leptin/adiponectin ratio serves as an indicator of subclinical atherosclerosis. (ii) Pharmacological administration of leptin can increase blood pressure in patients with human leptin deficiency syndrome. (iii) Therapeutic investigations for hyperleptinemia are still under investigation.

Chemirin	Increasing the expression of endothelial adhesion molecules (ICAM-1 and E-selectin) that share in the early steps of forming an atherosclerotic plaque.	Pharmacological inhibition of omentin may slow the pathogenic process of atherosclerosis.

Omentin	(i) Omentin has a NO-mediated vasodilator effect that can antagonize TNF-*α* mediated vasoconstriction. (ii) Inhibitory effect on smooth muscle migration and proliferation.	Potentiation of the molecular effects of omentin should be considered by pharmacological administration of its agonists or increasing the expression of its receptors.

Resistin	Promoting smooth muscle cells proliferation and migration and increasing the synthesis of prothrombotic tissue factor (TF) in human coronary cells.	It can serve as a prognostic factor for the severity of unstable angina and atherosclerosis.

Visfatin	Playing a significant role in plaque destabilization of unstable carotid and coronary atherosclerosis.	It can serve as a prognostic factor for cardiovascular mortality.

Apelin	Positive inotropic effect on the heart and exerting a relaxing effect on the aortic wall.	It may serve as an angiotensin II homologue, with a relaxing effect on the aortic wall.

## Abbreviations

A-FABP: Adipocyte fatty acid binding protein
AMPK: AMP-activated protein kinase
CRP: C-reactive protein
ECs: Endothelial cells
ICAM: Intercellular adhesion molecule-1
JNK: C-Jun N-terminal kinase
IR: Insulin resistance
MCP-I: Monocyte chemotactic protein 1
NF-kB: Nuclear factor-kappa B
PAI: Plasminogen activator inhibitor
PAVT: Perivascular adipose tissue
PPAR: Peroxisome proliferator-activated receptor
RBP-4: Retinol binding protein-4
ROS: Reactive oxygen species.
